# Local Thyroid Hormone Action in Brain Development

**DOI:** 10.3390/ijms241512352

**Published:** 2023-08-02

**Authors:** Andrea Alcaide Martin, Steffen Mayerl

**Affiliations:** Department of Endocrinology Diabetes & Metabolism, University Hospital Essen, University of Duisburg-Essen, Hufelandstraße 55, 45147 Essen, Germany

**Keywords:** thyroid hormone, T3, T4, glutamate, GABA, acetylcholine, dopamine

## Abstract

Proper brain development essentially depends on the timed availability of sufficient amounts of thyroid hormone (TH). This, in turn, necessitates a tightly regulated expression of TH signaling components such as TH transporters, deiodinases, and TH receptors in a brain region- and cell-specific manner from early developmental stages onwards. Abnormal TH levels during critical stages, as well as mutations in TH signaling components that alter the global and/or local thyroidal state, result in detrimental consequences for brain development and neurological functions that involve alterations in central neurotransmitter systems. Thus, the question as to how TH signaling is implicated in the development and maturation of different neurotransmitter and neuromodulator systems has gained increasing attention. In this review, we first summarize the current knowledge on the regulation of TH signaling components during brain development. We then present recent advances in our understanding on how altered TH signaling compromises the development of cortical glutamatergic neurons, inhibitory GABAergic interneurons, cholinergic and dopaminergic neurons. Thereby, we highlight novel mechanistic insights and point out open questions in this evolving research field.

## 1. Introduction

Thyroid hormone (TH) is absolutely essential for normal brain development, as it regulates critical processes such as precursor cell differentiation, migration, maturation, functional integration of neurons, synaptogenesis, and neurotransmitter synthesis [1,2,3,4]. However, rather than inducing processes per se, TH acts as a timing signal that coordinates and aligns the parallel development of different neuronal systems and associated glia cells [5]. Temporally, the requirement for TH varies with the developmental stage. A time window that is especially sensitive towards the presence of TH has been identified, ranging from late fetal stages to postnatal weeks 3–4 in rodents and the first postnatal months in humans [2,5]. The absence of TH during this critical time window has dramatic consequences for brain development by desynchronizing developmental sequences that will manifest in permanent structural and functional abnormalities. TH excess, on the other hand, is similarly detrimental due to premature differentiation events and disrupted developmental chronologies. Thus, much research has been focused on perinatal and early postnatal alterations in TH levels and their consequences for brain development and CNS morphology and function in adult stages, while much less is known about the equally important impact of TH on prenatal development [6,7].

TH and TH signaling components as a proxy for TH action have been unraveled in the embryonic brain at very early stages. T3 (3,3′,5-triiodothyronine) and its receptors have been detected in the human brain from the 10th gestational week (GW10) onwards—hence, even before the onset of endogenous fetal thyroid gland function in the second trimester in humans and at embryonic day 17 (E17)-E18 in the rat [5,6,8,9]. Thus, TH acts on brain development already when the fetus fully depends on maternal TH supply. Consequently, impaired maternal thyroid function compromises embryonic brain development and culminates in a low IQ, cognitive impairments, or behavioral problems such as attention deficit hyperactivity disorder in the offspring [10,11,12,13,14]. Moreover, maternal hypothyroidism has been linked to neurological disorders in children, including autism spectrum disorders, schizophrenia, anxiety, increased seizure susceptibility, and epilepsy. Maternal hyperthyroidism, likewise, results in an increased risk of developing epilepsy for the child [11,12].

Many of these neurobehavioral problems are strongly reminiscent of pathological alterations in neurotransmitter systems. Disruptions in the development of the inhibitory GABAergic system, for instance, also result in an increased seizure susceptibility and epilepsy in the offspring [15]. Therefore, it is not surprising that intensive research over the last decades has revealed a wealth of alterations in critical neurotransmitter systems in the CNS following an abnormal thyroidal state. However, most of these revelations remained rather superficial. As we discuss later in this review, the detailed importance of TH signaling components such as TH transporters, deiodinases, and TH receptors (TRs), as well as the exact changes and underlying abnormalities in the development of different neurotransmitter systems following altered TH levels in the CNS, are just beginning to emerge.

## 2. Regulation of TH Action in the Brain from Development to Adulthood

### 2.1. TH Uptake across Brain Barriers

With the closure of the neural tube, which occurs between E8.5 and E10 in mice and between E19 and E29 in humans [16,17], a compartmentalization process is initiated that restricts the access to the enclosed space and the neural progenitor cells lining it. This, in turn, necessitates the establishment of transport mechanisms to enable the flux of substances critical for later stages of brain development. TH represents such a factor that needs to be taken up from the circulation and transported to the inner compartment across forming barriers. In the developing murine brain, T4 (thyroxine; 3,3′,5,5′-tetraiodothyronine) has been detected as early as E16.5, while T3 was observed from at least E18.5 onwards [1,18,19,20]. However, whether this rather late-stage detection simply reflects technical challenges, such as a limited detection sensitivity, remains to be seen. Indeed, the expression of deiodinases and TH receptors as a proxy for TH tissue sensitivity was noted at much earlier stages. Consequently, the TH supply to the developing brain across barriers has to be guaranteed from these early stages of brain development onwards.

One barrier of utmost importance is the blood–brain barrier (BBB), which, in its mature state, is formed by endothelial cells and pericytes and covered by astrocytic endfeet [21]. Brain vascularization starts when neural tube closure is still in progress, and the most precocious performant vessels sprouting into the developing murine CNS tissue have been described as early as E9.5 to E10.5 [22,23]. These newly formed blood vessels do not form a tight barrier but are initially rather leaky, and a primitive BBB can be observed around E15 in mice [21]. Paralleling the process of BBB formation and, thus, a reduction in blood vessel leakiness, the T4 transporter Oatp1c1 (organic anion transporting polypeptide 1c1) becomes expressed in murine blood-vessel-like structures as early as E14.5 [24]. Thereafter, prominent Oatp1c1 expression has been observed throughout development and in adulthood in rodent brain endothelial cells at both the luminal and abluminal membrane [25,26,27,28,29]. In contrast, in the developing human brain, only very low expression levels of OATP1C1 were found in BBB endothelial cells from GW32 onwards [30]. A reciprocal species-specific difference was noticed for Mct8 (monocarboxylate transporter 8) that is not present in murine blood vessels before birth but was observed in human BBB endothelial cells from GW14 to GW38 [24,25,30,31]. In the adult CNS, however, pronounced endothelial Mct8 staining was observed in both humans and mice [25,27,32]. Among other known TH transporters, a prominent capillary staining in the adult brain has also been noticed for Lat1 (L-type amino acid transporter 1) [33,34,35]. In in situ hybridization experiments, Lat1 mRNA was observed already in the murine neural tube from at least E8.5 onwards, as well as in arising fore- and hindbrain structures, while in the developing chicken cerebellum, Lat1 transcripts could be mapped to brain capillaries [36,37].

Like the BBB, the blood–cerebrospinal fluid barrier (BCSFB) formed by the choroid plexus (ChP) represents a second barrier that restricts the access to the CNS. In its mature state, the ChP consists of fenestrated blood vessels, while the barrier proper is established by ChP epithelial cells that arise from the neuroepithelium [38]. There are, in total, four choroid plexi: two in the lateral ventricles, one in the third, and a last one in the fourth ventricle [39]. During neurodevelopment, the choroid plexus may play a critical yet understudied role as it generates cerebrospinal fluid (CSF) and, thus, the pressure needed for brain organization, as well as for its transport function, that downstream affects neural stem cells lining the ventricle walls and that are, therefore, directly exposed to compounds transported across the BCSFB [39]. Choroid plexus precursor cells are defined following neural tube closure and begin to emerge in the lateral ventricles from E11 onwards in mice [40]. To this end, a part of the dorsal midline invaginates, giving rise to the choroid plexus that expresses the TH transporter MCT8 already at very early stages, from at least E8 in chicken, E12.5 in mice, and GW14 onwards in humans [24,30,41]. Likewise, OATP1C1 is present at the BCSFB from at least E8 in chicken, E14.5 in mice, and GW32 in human embryos onwards. The spatiotemporal expression pattern of other TH transporters is less well defined. Using in situ hybridization, the L-type amino acid transporter 2 (Lat2) and the monocarboxylate transporter 10 (Mct10) have been observed in the murine ChP at postnatal stages, though their distribution in the embryonic mouse brain remains elusive [33]. The ChP also expresses high levels of the TH binding protein Transthyretin (Ttr), which can be detected from E11 onwards in mice and serves as a marker for the ChP [39]. Ttr represents the only TH binding protein in the CSF and constitutes up to 20% of its entire protein content [42].

In addition to these classical barriers, recent analyses on human embryonic tissue derived from GW14-38 identified two additional barriers: the outer cerebrospinal fluid–brain barrier between pial cells and basal end-feet of radial glia cells and the inner cerebrospinal fluid–brain barrier formed by neuroepithelial cells lining the ventricular system [30]. This latter barrier disappears later when neuroepithelial cells differentiate into radial glia cells. Hence, these observations put a new focus on radial glia cells in the regulation of local TH availability, which is further emphasized by the expression of MCT8 and OATP1C1 in this cell type, peaking at GW20 in the human fetal brain.

Together, recent studies have highlighted that our understanding as to when and where TH is taken up into the brain is far from complete, and ongoing research may reveal even more uptake routes and proteins involved.

### 2.2. Central Conversion of TH

Although the prohormone T4 can bind to TRs, it does so with less affinity than the more receptor-active form T3. In the brain, the T4-to-T3 conversion is catalyzed by type 2 deiodinase (Dio2), and early studies in rats have shown that roughly 80% of the total T3 pool in the CNS is generated this way, emphasizing the outstanding importance of Dio2 [43]. Dio2 activity is already detectable during early stages of brain development and even before the onset of fetal thyroid gland function [44,45,46,47]. Thereby, a pronounced ontogenic increase in fetal brain Dio2 levels can be observed with a four-fold elevation between E17 and E22 in rats [47]. Likewise, in human brain development, an increase in Dio2 activity that correlated with a surge in brain T3 content was seen until gestational week 20 [46].

In situ hybridization studies on P15 rat brain tissue mapped Dio2 expression to astrocytes and tanycytes lining the third ventricle [2,48]. Thus, as astrocytes are part of the BBB, astrocytic Dio2 is well-positioned to directly sense and convert T4 taken up across the BBB. In tanycytes, Dio2 is thought to contribute to the T3 supply to hypothalamic nuclei and may even be involved in the feedback regulation to hypothalamic paraventricular nucleus-residing TRH-expressing neurons, though the final proof for the latter concept is still pending [49].

The cellular distribution in the postnatal brain, however, does not necessarily reflect the picture seen during embryonic development. As an example, although Dio2 in the chicken brain is expressed in adult tanycytes, it was not detected there during embryonic stages [45]. In more recent studies, Dio2 mRNA expression was further noted in blood vessels in chicken embryonic brain sections derived from E8 to E18 [41]. In addition, Dio2 transcript was unraveled at the meninges, ependymal layer of the lateral ventricle, and choroid plexus of perinatal mice [18]. Together, these latest studies point to a yet understudied participation of Dio2 in the local regulation of TH availability and activity directly at brain barriers during early development.

Similarly, type 3 deiodinase (Dio3) harbors an important role for central TH metabolism by inactivating T4 and T3 to rT3 and T2, respectively [8,50]. In the postnatal brain, Dio3 presents with a prominent neuronal expression pattern [51,52]. Though Dio3 activity is relatively high at this stage, an even more pronounced activity is observed in the embryonic CNS when Dio3 is critical to protect the brain from the deleterious effects of excessive TH concentrations [50]. This further aligns with a broader expression pattern, as Dio3 mRNA was detected in the chicken ChP from E8 to the young-hatchling stage [41]. Over this time course, an incremental decline in brain Dio3 activity was reported [53]. Recent RNA sequencing studies in mice at E13.5 and E18.5 further advanced the idea that Dio3 is critical for the proper chronology of developmental events [54].

### 2.3. Thyroid Hormone Receptors Mediate TH Action

TH executes a multitude of genomic actions by binding to thyroid hormone receptors that essentially are ligand-dependent transcription factors bound constitutively to TH-responsive elements of T3 target genes [55]. TRs are encoded by two genes, THRA/TRα and THRB/TRβ, from which a number of different protein isoforms are generated by alternative transcription start sites or alternative splicing [56,57]. Thereby, TRα1, TRβ1, and TRβ2 are the main TH-binding isoforms. More recently, non-canonical actions of TH either by binding to cytosolic TRs and activating the phosphatidylinositol-3-kinase pathway or through the binding of T4 to the integrin αvβ3 membrane receptor and downstream activation of MAPK signaling have gained increased attention [42,58].

In the embryonic mouse brain, T3 binding was detected at E15.5 and, thus, earlier than the onset of fetal thyroid gland function [59]. However, technical challenges may mask an even earlier impact of TR activity on brain development. Along this line, TRα1 transcript was already found in the rat neural tube at E11.5, increased in the telencephalon around E13.5, and has surged in the developing cortex and hippocampus by E15.5 [60,61]. In comparison, TRβ1 mRNA was observed later and more distinctly from E17.5 onwards in the developing striatum, hippocampus, and neocortex in a pattern that indicated expression in proliferating neuroblasts. Both TRα1 and TRβ1 mRNA were also detected in the developing human brain from GW8 onwards and, thus, at a time when the fetus depends on maternal TH supply [62].

Postnatally, TRα1 expression peaked in the cerebellum, cerebral cortex, hippocampus, striatum, and olfactory bulb in the first three weeks of life and declined in adulthood [6,61,63]. Analysis of TRα1-GFP reporter mice further confirmed the presence of TRα1 in virtually all neurons in the adult brain [64]. TRβ1 expression peaked in the early postnatal cerebral cortex, hippocampus, striatum, and olfactory bulb and plateaued in adulthood at a lower expression level [6,61,63,65]. TRβ2, in contrast, is present in the paraventricular hypothalamic nucleus and the pituitary and, thus, is implicated in the regulation of the hypothalamus–pituitary–thyroid axis [66]. Importantly, while TRα1 is the main TR isoform in murine neurons, human neurons harbor significantly more TRβ; however, the consequences of this species-specific difference need to be further investigated [67].

All these investigations on the spatiotemporal expression profile of TH transporters, deiodinases, and TRs point to an early impact of TH signaling during critical phases of fetal brain development. We now discuss how proper TH signaling governs the development of neurotransmitter systems from early developmental stages onwards.

## 3. TH and Excitatory Glutamatergic Neurons

Glutamate is the major excitatory neurotransmitter in the brain. It is utilized by a variety of principle neurons, such as thalamic projecting neurons, hippocampal and cerebellar granule cell neurons, or pyramidal cells in the hippocampus and cerebral cortex. The vastness of its projection network is reflected by the observation that glutamatergic synapses constitute up to 80% of all synapses on a dendritic tree of neocortical or hippocampal cornu ammonis (CA1 and CA3) pyramidal neurons [68]. Glutamatergic neurons are involved in a myriad of physiological functions, such as learning and memory, cognition, awareness, emotional regulation, and other higher cognitive functions. Conversely, the dysfunction of glutamatergic neurons has been implicated in many CNS disorders, including Huntington and Alzheimer disease, epilepsy, anxiety, depression, and schizophrenia [69].

Over the last decades, a negative correlation between TH and cortical glutamate levels has been unraveled. Adult-onset hypothyroid patients presented with increased glutamate levels in the posterior cingulate cortex, while adult-onset hyperthyroidism resulted in diminished glutamate concentrations, as detected by magnetic resonance spectroscopy [70,71]. This correlation may be explained by a well-known effect of thyroid hormone on astrocytes, in which T3 induces the expression of the glutamate transporters Glast and Glt1, thereby accelerating the clearance of glutamate (Supplementary Table S1) [72]. Of note, abnormal TH and glutamate levels can manifest in similar neurological symptoms and mood disorders. Both adult-onset hypothyroidism and hyperthyroidism may result in depression, anxiety, and impaired cognition [73]. Likewise, decreased glutamatergic metabolite levels in the medial prefrontal cortex, as well as excessive glutamate levels in the circulation and the brain, were linked to depressive and bipolar disorders [74,75,76]. However, whether there is a direct link between the two systems in these medical conditions remains to be seen, and existing data need to be interpreted with caution due to the complexity of the disorders.

**Figure 1:**
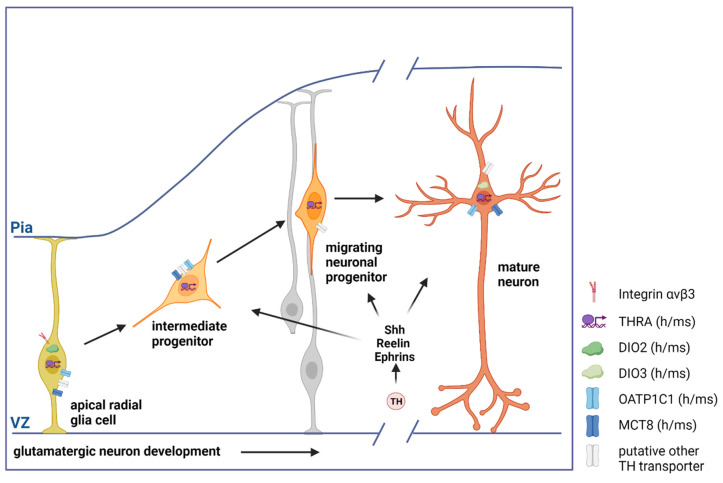
Schematic representation of different stages of cortical glutamatergic pyramidal cell development. Apical radial glia cells give rise to intermediate progenitors, which transform into neuronal progenitors that migrate along the radial glia scaffold to reach their destined position. In the developing cortical plate, progenitors mature into pyramidal cells, the dominant neuronal cell type in the cerebral cortex, which release the neurotransmitter glutamate. Expression of TH transporters, deiodinases, and TRs is depicted as known for the human and mouse cerebral cortex. TH-regulated factors such as Shh, Reelin, and Ephrins have been implicated in the regulation of corticogenesis. Gray-colored cells indicate radial glia cells (apical and basal) that provide the fiber scaffold for progenitor migration. VZ—ventricular zone. Created with BioRender.com.

The question as to how TH regulates glutamatergic neuron generation, differentiation, maturation, and function is gaining attention. In this respect, a major focus lies on cerebral cortex pyramidal cell development and the process of corticogenesis that occurs between E11 and E18 in rats and between gestational weeks 5 and 25 in humans [6,67]. This process starts with the asymmetric division of apical radial glia cells (aRGCs), pluripotent stem cells residing in the ventricular zone of the telencephalon (Figure 1) [77,78]. These aRGCs either generate neurons directly (direct neurogenesis) to form an initial preplate structure, or indirectly via cycling intermediate progenitors that populate the intermediate zone (indirect neurogenesis). Moreover, outer radial glia cells in the subventricular zone which have lost their apical attachment to the ventricular surface contribute to indirect neurogenesis. In evolutionary terms, indirect neurogenesis has an increasing impact with higher complexity and folding of the cerebral cortex [77,79]. Newly generated neuronal progenitor cells then migrate radially towards the pia surface and settle more superficially the later they are born, thus forming the different cortical layers in an inside-out pattern. Recent single-cell RNA sequencing studies demonstrated that fate acquisition occurs as a combination of pre-specification and progressive fate restriction involving internal and external cues to a varying degree [77].

TH represents such a critical external cue, as demonstrated by the observation that even modest maternal hypothyroidism during fetal neocorticogenesis has dramatic consequences for the offspring culminating in a pronounced reduction of cortical thickness [14,67,80]. Firstly, this has been attributed to compromised cell-cycle kinetics that result in a diminished progenitor pool, which in turn primarily affects indirect neurogenesis and the proper formation of the outer cortical layers [80]. Secondly, maternal hypothyroidism interferes with radial migration along the cortical radial glia fiber scaffold, causing neuronal misplacement and altered circuitry [67]. Key molecules that are thought to mediate the effects of altered TH signaling on neocorticogenesis are sonic hedgehog (Shh), Reelin, and Ephrins, which are well-characterized morphogens and regulators of migration, respectively. These factors, however, are extrinsically provided to developing pyramidal cell neurons, while a direct action of TH in radial glia cells, intermediate progenitors, or pyramidal cell neurons remains to be shown.

In this respect, it is interesting to note that progenitors in the ventricular zone express TRα1, Dio2, and high levels of Mct8 [30,80]. The advent of single-cell sequencing studies has provided a better cell-type resolution, demonstrating DIO2 and OATP1C1 transcripts in outer radial glia cells, thus rendering them effectively a major source for T3 in the developing human neocortex (Figure 1) [78,81]. In these experiments, low OATP1C1 and high MCT8 mRNA levels were further observed in intermediate progenitors, while MCT8 transcripts were detected in neurons, and THRA expression was found rather ubiquitously. More recently, the presence of MCT8 and OATP1C1 on the protein level was highlighted in mature pyramidal neurons of the human and macaque motor cortex by immuno-staining [31]. The impact of these TH-signaling regulators on neocorticogenesis was further substantiated by studies in genetically modified mice. Both Mct8/Oatp1c1 global double knockout and TRα1 mutant mice in which T3 binding capacity is reduced to 10% (TRα1-R384C mice) exhibit a thinning of the cerebral cortex that primarily affects the upper layers [82,83]. In another line of evidence, Vancamp and co-authors induced Mct8 knockdown in neural progenitors of the chicken optic tectum, a model for the mammalian cerebral cortex, using an RNAi vector approach [84]. In agreement with the murine data, Mct8 knockdown resulted in disrupted cell-cycle kinetics, compromised progenitor pool expansion, altered migration, and reduced thickness of the optic tectum. Together, these data demonstrate that Mct8/TRα1-mediated genomic TH signaling directly affects progenitors in the developing cerebral cortex.

In addition to intracellular events that are enabled by Mct8-mediated TH uptake, non-genomic TH action through the cell-surface T4 receptor integrin αvβ3 on apical and basal progenitors further contributes to the expansion of the progenitor pool in the subventricular zone [85]. Targeted receptor activation induces progenitor cell cycling and a surge in intermediate progenitors, while the blocking of TH binding abolished pool expansion.

Although final proof is still missing, all indications suggest a cell-autonomous function of TH signaling in radial glia cells and, thus, the pyramidal cell lineage. Future conditional knockout approaches will certainly help to shed new light on this exciting field of research.

## 4. TH and Inhibitory GABAergic Interneurons

GABA, the main inhibitory neurotransmitter in the CNS, is synthesized from glutamate by the activity of glutamate decarboxylase (GAD). GABA is utilized by a variety of neuronal subtypes, such as cerebellar Purkinje cells, striatal medium spiny neurons, or inhibitory interneurons, which we focus on in this review. GABAergic interneurons represent a highly heterogeneous group of neurons that, according to the most widely used classification system, are categorized into three major subgroups with non-overlapping marker expression: parvalbumin (PV), somatostatin (SST), and serotonin receptor 3A (5-HTR3A) positive interneurons [86,87,88]. Other markers, such as calretinin (CR), neuropeptide Y (NPY), vasointestinal peptide (VIP), or cholecystokinin (CCK), exhibit an overlapping expression pattern within the three major populations. Impairments in the generation, migration, integration, or synaptogenesis of interneurons have been associated with a number of psychiatric and neurological disorders, such as schizophrenia, epilepsy, or autism spectrum disorders [89,90].

Several lines of evidence suggest a direct interplay between TH and the GABAergic system. Developmental hypothyroidism is known to reduce central GABA levels, e.g., by affecting the GABA producing enzyme GAD [89,91]. Controversial results were obtained for adult-onset hypothyroidism that, in rats, culminated in increased central GABA content [89,92,93]. In contrast, in a recent longitudinal magnetic resonance spectroscopy study, reduced cortical GABA levels were observed in adult-onset hypothyroid patients, which were normalized upon L-T4 therapy [94]. Despite species-specific differences, caution is required as to the origin of the GABA signal, as GAD activity and GABA content may be differentially regulated in GABAergic interneurons and, e.g., medium spiny neurons in the striatum.

**Figure 2:**
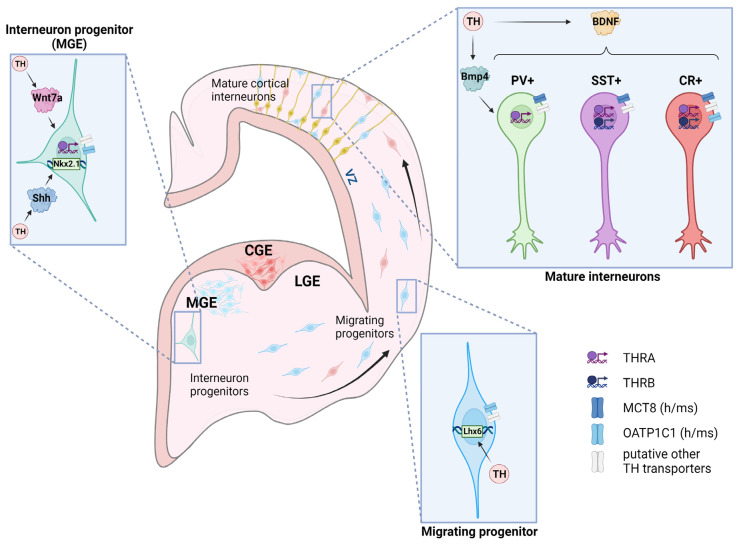
TH’s effects on the development of inhibitory GABAergic interneurons. Interneuron progenitors are generated in the ganglionic eminences, with the medial ganglionic eminence (MGE) giving rise to the PV+ and SST+ subtypes, whereas CR+ interneurons are born in the caudal ganglionic eminence (CGE). Progenitor development is regulated by T3 target genes such as Shh, which is critical for the maintenance of Nkx2.1 expression. Likewise, T3-regulated Wnt components may influence early interneuron progenitor development. Progenitors migrate out of the ganglionic eminence to populate brain areas such as the forming cerebral cortex. Lhx6 is implicated in cellular migration and is itself TH sensitive and downstream of the T3/Shh/Nkx2.1 pathway. Interneuron maturation is further governed by TH-regulated Bdnf and Bmp4. Mature interneurons can be distinguished according to different classification criteria, such as the expression of marker proteins PV, SST, or CR. Expression of TH transporters, deiodinases, and TRs is depicted as known from murine and human studies. LGE—lateral ganglionic eminence; VZ—ventricular zone. Created with BioRender.com.

In humans, interneuron neurogenesis takes place between GW10 and GW25, whereas in mice, the bulk of interneurons is generated between E12.5 and E16.5 in the ganglionic eminences of the ventral telencephalon in a temporally and spatially tightly regulated process [15,95]. Regarding spatial differences, PV and SST interneurons are born in the medial ganglionic eminence (MGE), whereby ventral parts adjacent to the preoptic area are biased towards generating PV+ interneurons and dorsal regions predominantly give rise to the SST+ subtype (Figure 2) [86,95,96,97]. The majority of CR+ interneurons, in contrast, is generated in the caudal ganglionic eminence (CGE) posterior to the MGE. Temporally, the peak in SST+ interneuron neurogenesis occurs earlier than the peak in the PV+ subtype, though both are derived from the same area, whereas CGE-derived interneurons are generated slightly later than those originating from the MGE. In order to reach their destined location in the neocortex, interneuron progenitors next migrate tangentially through the developing neocortex, where they receive layer information, switch to radial migration, and eventually integrate into the forming neuronal network.

Regarding the mechanistic bases for this interplay in inhibitory interneurons, in recent years a remarkable impact of TH on all stages of inhibitory interneuron development has been elucidated. Interneuron neurogenesis critically depends on the presence of thyroid hormone, and, interestingly, the PV+ subtype appears to be particularly sensitive towards TH. Studies in rats that were exposed to chemically induced hypothyroidism during pregnancy unveiled a long-lasting reduced PV immuno-staining in the offspring in a dose-dependent manner that could be rescued by additional T4 application [98,99]. It was suggested that the severity and onset of the maternal hypothyroidism correlates with the effects on the PV+ inhibitory system. Even modest reductions in maternal TH may therefore hamper proper PV interneuron formation and innervation [14,98,100]. Similar observations of impaired PV+ neuronal maturation were made in a growth-retarded (grt) mouse model, in Mct8/Oatp1c1dko mice that present with a severely TH-deficient brain, in TRα1-R384C mice, or in mice that expressed a dominant negative form of TRα1 in all interneurons [82,83,101,102]. To date, the question as to whether hypothyroidism indeed reduces the number of PV+ cells or if the PV marker is just absent from that population is still elusive. A decreased number of PV+ interneurons is, however, frequently paralleled by an increase in the SST+ and CR+ subtypes, arguing for more complex changes, such as altered fate decisions [24,83,101,102]. Attempts to reverse these alterations by early postnatal T3 application have yielded controversial results in different mouse models but point to a critical TH-sensitive period, which encompasses the first few postnatal days, before pathological changes become persistent. Whereas T3 administration between P0 and P20 was able to restore a normal PV interneuron development in grt mice, TH injections between P11 and P13 failed to achieve a similar amelioration in TRα1-R384C mice [83,101]. This, together with the observation of combined cell-autonomous and non-cell-autonomous TH action, argues for multiple regulatory effects of TH on different steps in interneuron development [24,102].

As one such step, interneuron fate specification is a complex process that depends on external and internal signaling cues. Fate acquisition of MGE-derived interneurons occurs following their exit from the cell cycle, and, thus, many MGE progenitors are bipotential [103]. As one example, the mode of cell division, i.e., apical versus basal, determines whether the SST+ or PV+ subtype, respectively, is generated. Both, however, depend on the presence of the transcription factor Nkx2.1, and the deletion of Nkx2.1 induces a fate shift towards the generation of CR+ interneurons in the MGE [15,104]. For proper specification of MGE progenitors, fibroblast growth factor (Fgf) and Shh signaling are mandatory [105]. As a well-studied example, downregulation of Shh, a critical morphogen and key regulator of neural development, or ablation of the Shh-receptor Smoothened (Smo) in the MGE results in insufficient maintenance of Nkx2.1 and the genesis of CR+ interneurons in the MGE on the expense of PV+ and SST+ subtypes [97,106]. Shh is a well-established T3 target gene, and therefore its transcript levels, just like the mRNA levels of its receptors Smo and Patched (Ptc), were decreased in rat offspring in a model of maternal hypothyroidism [107]. This puts T3 at the top of a signaling cascade that eventually governs the generation of various interneuronal subtypes (Figure 2). In support of this idea, the specific activation of a dominant negative form of TRα1 in GABAergic interneurons resulted in reduced Shh expression, thus highlighting a cell-autonomous TRα1 function [102]. Likewise, we recently observed reduced Shh, Smo, Ptc1, and Nkx2.1 transcript expression in Mct8/Oatp1c1 dko mice at E12.5 [24]. These alterations were, however, not replicated in mouse mutants with conditional deletion of Mct8/Oatp1c1 in Nkx2.1-positive progenitors in agreement with absent Mct8 and Oatp1c1 protein staining in these progenitors, thus excluding the possibility of a cell-autonomous importance of both TH transporters. Hence, the question as to how TH signaling is regulated in MGE progenitors, or in other words, which TH transporters, deiodinases, and, possibly, other TRs than TRα1 are involved, awaits further answers.

In addition, MGE progenitor proliferation and fate specification require proper Wnt signaling. Acting through downstream activation of Notch signaling, Wnt ligands have recently been shown to maintain the MGE identity and to stimulate progenitor pool expansion in an in vitro assay using human ESC-derived MGE progenitors, while inactivation of the Wnt-signaling pathway induced neuronal differentiation [108]. Among the multiple Wnt ligands that were upregulated in MGE progenitors, Wnt7a was most strongly induced. Interestingly, Wnt7a is itself TH-sensitive [109], and thus, a yet unknown regulatory influence of TH on MGE progenitor proliferation can be hypothesized (Figure 2; Supplementary Table S1).

TH has further been suggested to govern migration events, making it especially important for interneuron progenitors that have to traverse long distances before they eventually integrate at their predestined position. Consequently, developmental hypothyroidism interferes with the proper migration of immature interneurons. This phenomenon has most prominently been observed in rats that were exposed to maternal hypothyroidism during the late stages of embryonic development, with E19 to P2 being an exceptionally sensitive window towards a disruption in TH signaling [110,111,112]. In these studies, misplaced GABAergic neurons in subcortical band heterotopia (SBH) clusters were noticed whose areas correlated with the degree of maternal hypothyroidism. Such heterotopias in rodents and children have been linked to pathological alterations such as learning deficits or, in a severe state, epileptic seizures [98,110,111,113,114,115]. Recently, we revealed the presence of Oatp1c1 protein in MGE-derived progenitors in the cerebral cortex of P0 mice, which points to a cell-autonomous role of TH in interneuron migration/maturation [24]. Moreover, early transplant studies in mouse embryos derived from euthyroid and 1-methyl-2-mercaptoimidazole (MMI)-treated hypothyroid mice demonstrated that radial, but not tangential, migration of interneurons in the neocortex is affected [116]. Together, these results also suggest that cell-autonomous and non-cell-autonomous signals from the surrounding environment are altered in the hypothyroid embryonic cortex. As one example, the transcription factor Lhx6 is critical for tangentially migrating interneurons originating from the ventral telencephalon [117]. Lhx6 is situated downstream of Nkx2.1 and may thus be already compromised following a reduced Shh/Nkx2.1 pathway activation in the hypothyroid embryonic brain [95]. In addition, ChIP-on-chip analyses have demonstrated Lhx6 as a direct T3 target, positioning it as a potential key factor in the cell-autonomous regulation of interneuron migration by TH [118]. Bdnf (brain-derived neurotrophic factor), on the contrary, represents a non-cell-autonomous signaling cue that guides interneuron migration and influences their final differentiation and maturation. Its TH-dependency has been reported in various animal studies, and the loss of proper Bdnf signaling was linked to a downregulation of cortical interneuron markers [87,88,119,120]. Likewise, TH may impact the differentiation of at least a subset of interneurons through Bmp4. A rise in neocortical Bmp4 levels is paralleled by the appearance of PV interneurons in the cortex, while SST+ interneuron differentiation is inhibited [121]. Moreover, in the hypothyroid neonatal brain, a marked decrease in Bmp4 was correlated with aberrant PV interneuron development, as discussed above [122].

Though the spatiotemporal profile of TH-signaling regulators during GABAergic interneuron development remains largely elusive, recent analyses have begun to decipher their expression pattern in mature interneurons. In addition to the heterogeneous nature of interneurons, there appears to be species-specific differences between mice and primates. In the mouse, Mct8 protein was found in the PV+ but not CR+ subtype [24]. Along this line, immuno-histochemical profiling of TH transporters in cortical interneurons in the macaque cortex revealed MCT8 expression in PV+, as well as in SST+ interneurons, while OATP1C1 protein was observed in CR+ and Calbindin+ interneurons [31]. A transcriptomic analysis of human cerebral cortex samples highlighted the presence of THRA, THRB, and MCT8 transcripts in CR+ and SST+ interneurons, but the PV+ subtype was not investigated [78,81]. Accordingly, MCT8 was identified in CR+ and SST+ human cortical interneurons in recent immuno-staining approaches [31]. However, their impact on interneuron maturation and function remains unknown and needs to be addressed in subsequent studies.

The last decade has seen significant progress in our understanding of the cell-autonomous and indirect effects of TH on inhibitory interneuron development. Though some TH-signaling regulators have been critically associated with this, the picture is far from complete. A detailed spatiotemporal profile of these regulators in the different interneuron subtypes and developmental stages is still elusive, and so is the question as to how their expression and action can be linked to the observations summarized above.

## 5. TH and the Cholinergic System

Cholinergic neurons have been implicated in maintaining the excitation/inhibition balance within the CNS and the fine-tuning of a variety of CNS functions, such as sleep and wakefulness, locomotor activity, and cognition, including memory function and learning abilities [123,124,125]. Metabolically, the activity of the neurotransmitter acetylcholine (ACh) is regulated by the activity of two key enzymes: Choline-O-Acetyltransferase (ChAT), which synthetizes in a one-step reaction ACh from choline and acetyl-CoA, and Acetylcholine-Esterase (AChE), which catalyzes ACh’s hydrolysis, thus decreasing neurotransmitter availability [126].

The dependency of cholinergic neurotransmission on proper central TH levels was recognized a long time ago. The 6-propyl-2-thiouracil (PTU)-mediated induction of hypothyroidism in rats resulted in reduced ACh levels in the hippocampus [127]. Along this line, ChAT activity is decreased in the hypothyroid brain and is enhanced following the administration of TH (Supplementary Table S1) [128,129,130,131]. Controversial results were obtained for AChE, with some reports demonstrating downregulated AChE activity [127,132], while others reported an increased AChE enzymatic activity in the PTU-induced hypothyroid state in rats [133,134]. Likewise, T4 administration to rats caused a decreased AChE activity, whereas the administration of T3 in vitro resulted in an enhanced enzymatic activity, together with a stabilization of AChE mRNA [132,135]). These discrepancies may be explained by different treatment protocols, variations in the genetic background, or variations in methodical approaches. The majority of observations, however, converge on the idea of a positive correlation between the thyroidal state and cholinergic activity.

Beyond the activity of both key enzymes, little is known about the impact of TH on cholinergic neuron development, maintenance and physiology let alone their heterogeneity. In the adult brain, cholinergic neurons are found at two major sites: in the basal forebrain and the brainstem. In the latter area, cholinergic neurons are localized in the pedunculopontine tegmental nucleus (PPT) and the laterodorsal pontine tegmentum (LDT) from where they project mainly to the thalamus, ventral tegmental area and the basal forebrain cholinergic system [125,136,137]. Basal forebrain cholinergic nuclei include the medial septal nucleus, the vertical and horizontal part of the diagonal band of Broca, and the nucleus basalis of Meynert from which projections are sent to several cortical and hippocampal areas. As one example, ACh+ neurons in the medial septal nucleus project to the hippocampus and, thus, are thought to contribute to learning processes (septo-hippocampal pathway). The majority of basal forebrain cholinergic neurons is derived from the MGE, the preoptic area (POA), and the septal epithelium, while subsets are born in the pallium [125]. Recent genetic fate mapping and intersectional focal septal deletion studies have demonstrated an essential role for Nkx2.1 in the development of basal forebrain cholinergic neurons, as well as proper learning and memory function [138]. Likewise, cholinergic interneurons derived from the MGE, CGE, POA, and septal epithelium that populate the cortex and basal ganglia arise from Nkx2.1-positive progenitors that co-express the markers Isl1 and Lhx8 [125]. As we discussed in the previous chapter on the development of GABAergic interneurons, the maintenance of Nkx2.1 levels is influenced by the thyroidal state. Therefore, it is tempting to hypothesize that TH affects cholinergic projecting neuron and ACh+ interneuron development similarly from very early embryonic stages onwards. Along this line, in re-aggregated primary cultures derived from the E16 rat brain, T3 was found to enhance the differentiation of cholinergic neurons, hence again highlighting an early requirement of TH [139]. Details on directly participating TH transporters, deiodinases, and TRs, however, are still widely elusive though the involvement of TRβ1 has been suggested [140]. In addition, TH may regulate cholinergic neuron development indirectly via interaction with nerve growth factor, which also harbors an important role in basal forebrain neuron growth and maintenance [1,130].

Despite the unequivocal entanglement of the thyroidal and the cholinergic system in the CNS, surprisingly little is known about the underlying pathways and key mediators involved. Thus, many interesting observations are to be made in the future in this highly important and promising field of research.

## 6. TH and the Dopaminergic System

Although dopaminergic neurons extend a widespread network into the adult forebrain, they are localized to only a few distinct clusters in the olfactory bulb, hypothalamus, and, most importantly, in the mesencephalon [141,142,143]. In the latter structure, dopaminergic neurons are found in the substantia nigra pars compacta, an area that projects to the striatum (neostriatal pathway), as well as in the ventral tegmental area (VTA), which projects to the cerebral cortex and limbic structures (mesocortical and mesolimbic pathways, respectively). Dopaminergic neurons have been implicated in the control of movement, cognition, mood, and behavior, as well as in multiple neurological disorders, such as Parkinson disease, schizophrenia, or attention deficit hyperactivity disorder (ADHD) [143]. Biochemically, the neuromodulator dopamine is synthesized from tyrosine via the intermediate DOPA in a two-step reaction catalyzed by tyrosine hydroxylase and DOPA decarboxylase.

The thyroidal state is a well-known determinant of central dopamine concentrations, and some neurological aspects of aberrant TH levels are thought to be mediated through the midbrain dopaminergic (mDA) system. Induction of hypothyroidism by PTU administration to adult rats resulted in a rise in hypothalamic, cortical, and hippocampal dopamine levels [144], whereas neonatal hypothyroidism reduced dopamine-producing tyrosine hydroxylase and dopamine turnover in the striatum [91,145]. In the offspring of female mice, which were rendered hypothyroid by PTU treatment during pregnancy, a reduced mDA neuron formation was detected when analyzed at different pre- and postnatal time points, along with decreased tyrosine hydroxylase and dopamine transporter expression levels, as well as affected mDA neuron-related behaviors [145,146,147]. This aligns with early studies pointing to congenital hypothyroidism as a risk factor for reduced mDA neurons and associated movement disorders [148]. Experimental hyperthyroidism induced by daily T4 injections in young and adult rats resulted in increased dopamine levels in multiple brain areas [149]. Evidence supporting the interplay between the thyroidal and dopaminergic systems was further found in TRβ knockout mice. In these mice, the negative feedback regulation of TH on the hypothalamus–pituitary–thyroid axis is disrupted, leading to strongly elevated serum TH levels that subsequently affect the brain through TRα1, the chief TH receptor in the CNS, culminating in a central thyrotoxic state [6]. In turn, this may explain why TRβ knockout mice present with reduced dopamine turnover in the caudate putamen and, thus, higher dopamine levels that are thought to contribute to their ADHD-like phenotype [150].

During development, mDA neurons are derived from progenitors situated near the ventral midline of the neural tube floor plate around the cephalic flexure [141]. In mice, early mDA neuronal progenitors are generated between embryonic days E8.5 and E10.5 and acquire a dopaminergic fate between E10.5 and E12.5, followed by their terminal differentiation after E12.5. These processes are dependent on a variety of intrinsic and extrinsic factors that are expressed in a specific temporal and spatial sequence. Thereby, two organizers provide critical morphogens: the ventral floor plate organizer at the midbrain/hindbrain boundary secretes Shh, while the isthmic organizer provides Fgf8. Together, they establish a two-dimensional Cartesian coordinate system that specifies midbrain progenitors, including mDA progenitors [141].

Growing evidence from in vitro and in vivo studies demonstrates that TH is an important extrinsic factor that is already regulating mDA neuron formation during embryonic stages. While non-cell-autonomous effects of altered TH concentrations on the expression and gradients of morphogens from midbrain organizers remain to be investigated, a cell-autonomous TH-signaling component in mDA neuronal precursors has been identified. TH was shown to be a key factor in stimulating DA neuron formation from both primary mouse NSCs and rat neural precursor cells (NPCs), as well as from human ESCs-derived NPCs in vitro [146,151]. Investigating the underpinning molecular mechanisms, a TH/TRα1-dependent up-regulation of Otx2 (Orthodenticle Homeobox 2) has been unraveled [146]. Otx2, in turn, mediates the upregulation of Nurr1 and Ngn2, two factors that are implicated in the biogenesis of mDA neurons, and the silencing of Otx2 abolished the T3 effects on Nurr1 and Ngn2 levels and mDA neuron formation [146,152,153]. Subsequent experiments have shown that Otx2 is not a direct target of cell-autonomous TH signaling but involves the T3/TRα1-mediated upregulation of the calcium channel TRPC1 (transient receptor potential channel 1), the dominant TRPC channel in ventral midbrain NSCs, and an increase in cellular calcium signaling (Supplementary Table S1) [154]. Currently, the question as to how sufficient levels of T3 are provided locally to mDA neuron progenitors, i.e., which TH transporters are involved and how deiodinases are spatiotemporally regulated in this particular brain area during development, remains open.

## 7. Limitations

In our review, we focused on information about the general impact of an altered thyroidal state on the generation of selected types of neurons and neurotransmitter/neuromodulator systems in the brain. This summary is by far not complete as other systems such as the serotonergic or noradrenergic systems, for which even less information is available, have not been covered. Moreover, we did not discuss TH’s impact on the generation of synapses, the functional units of neurotransmission. TH is a key factor regulating dendritic morphology and synaptogenesis and governs the expression of various pre- and postsynaptic components, including munc18, syntaxin-1, synapsin-1, or synaptotagmin, directly or possibly through its effects on neurotrophins such as Bdnf [155,156,157,158]. However, as dendritic morphology varies between brain areas, the precise consequences of an altered thyroidal state on dendrite development and synaptogenesis may also be a function of the specific brain area, developmental stage, and neuron type. Likewise, changes in the levels of neurotransmitters measured in whole brains or macro-dissected brain areas may not necessarily reflect differences in the synapse. Hence, caution is needed to link brain-wide alterations in neurotransmitter levels following modulation of the thyroidal state to functional outcomes.

## 8. Concluding Remarks

The last decades have seen enormous progress in deciphering the impact of TH on brain development and function from very early embryonic stages onwards, in general, as well as on the formation, maturation, and maintenance of different neurotransmitter systems, in particular. For most systems, however, only the tip of the iceberg has been unraveled so far, and our review laid bare some tremendous gaps in our knowledge. Technical and methodical advances such as inducible conditional knockout and knock-in approaches, genetic-lineage tracing, or high-throughput sequencing have just started to be implemented. Thus, in future years, we can expect a much clearer picture of the local TH action in the (developing) brain and a much better disentanglement of the direct, cell-autonomous effects and indirect influences of TH.

## Data Availability

No new data were created or analyzed in this study. Data sharing is not applicable to this article.

## Supplementary Materials

The following supporting information can be downloaded at: https://www.mdpi.com/article/10.3390/ijms241512352/s1.
