# Supplementary material for: Local Thyroid Hormone Action in Brain Development
Source: Int J Mol Sci. 2023 Aug 2;24(15):12352. doi: 10.3390/ijms241512352 (PMC10418487; doi:10.3390/ijms241512352)
Supplement: Supplementary file 1 [file ijms-24-12352-s001.zip › ijms-2515371-supplementary.pdf]

Table S1: Gene products involved in mediating TH effects on the development of selected neurotransmitter systems

Neuro-transmitter system	TH regulated gene	Product	Relevance in neurotransmission	References
Glutamatergic system	Slc1a3	Glast	Glutamate transporter involved in glutamate re-uptake	Mendes-de-Aguiar et al., 2008
	Slc1a2	Glt-1	Glutamate transporter involved in glutamate re-uptake	Mendes-de-Aguiar et al., 2008
	Reln	Reelin	Glycoprotein involved in progenitor migration and positioning during neocorticogenesis	Alvarez-Dolado et al., 1999
GABAergic system	Pvalb	PV	Ca^{+2} binding protein expressed by one of the most abundant GABAergic interneuron populations	Berbel et al., 1996, Auso et al., 2004, Gilbert et al., 2007, Wallis et al., 2008, Uchida et al., 2014, Mayerl et al., 2014, Gilbert et al., 2020, Richard et al., 2020
	SST	SST	Peptide hormone used as a marker of a subset of GABAergic interneurons	Uchida et al., 2014, Richard et al., 2020, Mayerl et al., 2022
	Calb2	CR	Ca^{+2} binding protein used as a marker of a subset of GABAergic interneurons	Wallis et al., 2008, Mayerl et al., 2014, Richard et al., 2020
	Nkx2.1	Nkx2.1	Transcription factor necessary for the generation of PV and SST interneurons in the MGE	Mayerl et al., 2022
	Shh	Sonic Hedgehog	Morphogen required for the maintenance of Nkx2.1 and interneuron neurogenesis in MGE, and neocorticogenesis	Desouza et al., 2011, Richard et al., 2020, Mayerl et al., 2022
	Smo	Smoothened	Frizzled Class Receptor	Desouza et al., 2011, Mayerl et al., 2022
	Ptch1	Ptc1	Shh receptor	Desouza et al., 2011, Mayerl et al., 2022
	Wnt7a	Wnt7a	Wnt ligand, possible role in MGE progenitor proliferation	Morte et al., 2018,
	Lhx6	Lhx6	Transcription factor involved in tangential migration of interneuron progenitors	Dong et al., 2009
	Bmp4	Bmp4	Morphogen implicated in interneuron development	Martel et al., 2002,
	Bdnf	Bdnf	Neurotrophic factor involved in migration and maturation of interneuron progenitors	Madhusudhan et al., 2022, Yajima et al., 2021, Jones et al., 1994

Cholinergic system	ChAT	Choline-O-Acetyltransferase	ACh synthesizing enzyme	Hefti et al., 1986, Gould and Butcher, 1989, Juarez de Ku et al., 1994, Sawin et al., 1998, Wang et al., 2015
	AChE	Acetylcholine-Esterase	Mediates ACh hydrolysis	Salvati et al., 1994, Puymirat et al., 1995, Carageorgiou et al., 2005, Wang et al., 2015, Cardoso et al., 2021
Dopaminergic system	TRPC1	TRPC1	Dominant Ca^{+2} channel in ventral midbrain NSCs	Chen et al., 2017
	Otx2	Otx2	Transcription factor involved in mDA neuron development	Chen et al., 2015
	Nr4a2	Nurr1	Transcription factor involved in mDA neuron formation	Chen et al., 2015
	Neurog2	Ngn2	Transcription factor involved in mDA neuron formation	Chen et al., 2015
